# The Spectrum of Motivations Behind Watching Mukbang Videos and Its Health Effects on Its Viewers: A Review

**DOI:** 10.7759/cureus.44392

**Published:** 2023-08-30

**Authors:** Sanskriti Sanskriti, Ishita Guglani, Shiv Joshi, Ashish Anjankar

**Affiliations:** 1 Department of Medicine, Jawaharlal Nehru Medical College, Datta Meghe Institute of Higher Education and Research, Wardha, IND; 2 Department of Community Medicine, Jawaharlal Nehru Medical College, Datta Meghe Institute of Higher Education and Research, Wardha, IND; 3 Department of Biochemistry, Jawaharlal Nehru Medical College, Datta Meghe Institute of Higher Education and Research, Wardha, IND

**Keywords:** eating disorders, obesity, aloneness, youtube, mukbang

## Abstract

Mukbang are videos in which individuals record themselves while eating a specific kind of food (typically food that is low in nutrition) and chit-chatting with the audience through virtual comments about their everyday lives. Since the beginning of this decade, mukbang videos have been gaining popularity all over the world, thanks to the growing popularity of online social media platforms, in particular YouTube. Since a limited number of studies have been carried out to investigate the motivations and effects of watching mukbang on its audience, the purpose of this review article is to analyze the existing body of research on mukbang and to shed light on myriads of the motivation behind watching mukbang videos and its health effects on its viewers. With the help of this review article, we want to bring attention to the importance of conducting high-quality research in this field so that medical professionals are aware of the motivations behind watching these videos and the conditions that may be associated with it. The motivations behind watching mukbang videos cannot be completely categorized into being positive or negative, since it depends on its usage by the viewers. Watching mukbang is significantly more prevalent among young adults. Mukbang offers digital commensality, entertainment, a para-social effect, escapism from real-life issues, and the opportunity for sexual use. On the other hand, excessive use of mukbang has been linked to a wide variety of health problems, including obesity and eating disorders. Since a limited amount of research has been conducted on mukbang, there is a pressing need to place emphasis on the phenomenon of mukbang, and clinicians should be made aware of mukbang in order to facilitate the diagnosis of conditions that are linked to it.

## Introduction and background

The idea that gave birth to the World Wide Web in the 1980s, which later became famously known as WWW, went on to irreversibly dominate the lives of those who use it in the current scenario [1-5]. Today, people use the Internet for a wide variety of tasks, each of which is determined by the requirements of the user, such as giving or taking advice, learning new languages, participating in dramatics, online gaming, online gambling, cooking, socializing, and many other activities [6-9]. Over the course of the last decade, the use of social media platforms such as Facebook, Instagram, Twitter, and YouTube, among others, has seen enormous growth in popularity [10,11]. It has been observed that YouTube is the platform that has the most users among the others [10]. One of the studies found that approximately 75% of adults and 94% of young adults in the United States of America spend up to three hours on YouTube on a weekly basis [12]. Studies have shown that problematic or excessive Internet use can cause marked functional derangement, which in turn lowers the quality of life of a human being [1-4,13]. It has been observed that a person will engage in such online activities when they are unable to satisfy their offline needs. These online activities offer the users instant gratification and, as a result, "escapism" from the realities of everyday life [7,9-11,13-16]. Mukbang is one of these trends that has gained popularity online in recent years. Mukbang is an online show in which a broadcast jockey eats food while interacting with viewers. It has become a popular form of entertainment because it can alleviate feelings of isolation by creating the impression of a communal meal, it can make one feel full without the consumption of potentially harmful foods, and it has a calming effect on the mind as well [9,17]. Despite this, many aspects of mukbang have been shown to have a negative impact on its audience, leading to issues such as obesity, overeating, multiple eating disorders, the belief that there is a negative correlation between the amount of food consumed and leanness, and poor table manners [17,18]. As a result, the purpose of this research is to conduct a literature review on mukbang and to focus attention on the numerous motivations behind watching mukbang and the health conditions that may be associated with it.

## Review

Origins and evolution of mukbang

This Korean word is a combination of two different Korean words: "meokneun" and "bangsong," which literally mean "eating" and "broadcast," respectively [6-9,13,17,19-23]. This word originated in South Korea in the late 2000s. During this activity, a broadcast streamer known as a mukbanger consumes large quantities of food, which is typically of a single type and may include fried chicken, ramen noodles, or rice cakes, all while interacting with viewers [8,9,22,23]. It has also been observed that the streamers have the potential to amplify a variety of sounds that occur during the act of eating, including the noises made by chewing loudly, biting into crispy fried chicken, and drinking soda [13].

The host or streamer will typically have a courteous and gentle manner of speaking to the viewers, and the streamers can frequently be found chatting with the audience during the online live eating show [9,22]. Although the mukbang trend was initially started in 2014 by South Korean mukbangers on the Korean video platform known as Afreeca TV, it soon spread rapidly to other parts of the world and became common on the social media platform YouTube, where it does not necessarily have to be live streamed [6-8,13,20,23,24]. Mukbang videos have gained a significant following on YouTube due to the fact that food is YouTube's most popular category [25]. Mukbang is a trend that has been gaining popularity rapidly, and as it does so, people from all different regions are adopting the practice of consuming food from a variety of different cuisines. Mukbangers who attract a significant number of viewers can make as much as $10,000 per month in today's market [22]. Mukbang and its constituents have been linked to a wide variety of psychological motivations making people watch it regularly, which has ultimately contributed to the rise in popularity of this practice.

The viewers of mukbang

The act of sharing a meal with other people, whether they be members of one's own family or friends, is regarded as one of the most significant ways of connecting and socializing with other beings in many different cultures around the world. The term "communal feasting" has its origins in a time period that spans approximately 12,000 years ago [6,26,27]. The people who watch live eating competitions, also known as mukbang, are typically solitary individuals. The negative emotion of loneliness in people who live apart from one another which they experience while eating is a major factor that has contributed to the rise in demand for eating shows which focus on binge eating [2-4,6-9,17,19-23,26,28]. According to the findings of a survey that was carried out in the United Kingdom, 15% of the viewers have not shared a meal with a member of their family in the preceding half year [26,29]. It has come to light in recent times that there has been a significant increase in the number of people who live by themselves; in the United States, the number of people who live alone has increased by a factor of five since the 1960s [26].

Motivations behind watching mukbang videos and its health effects on its viewers

It has become clear that mukbang is more than just a visual representation of the act of eating food on screen. Every single facet of mukbang contributes, in some way, to the experience that its viewers have. As a result, it is not known at this time whether or not the recently emerged trend of watching videos of people eating food that they have prepared themselves, known as mukbang, is beneficial or detrimental, or to what degree the former affects the latter [23].

**Figure 1:**
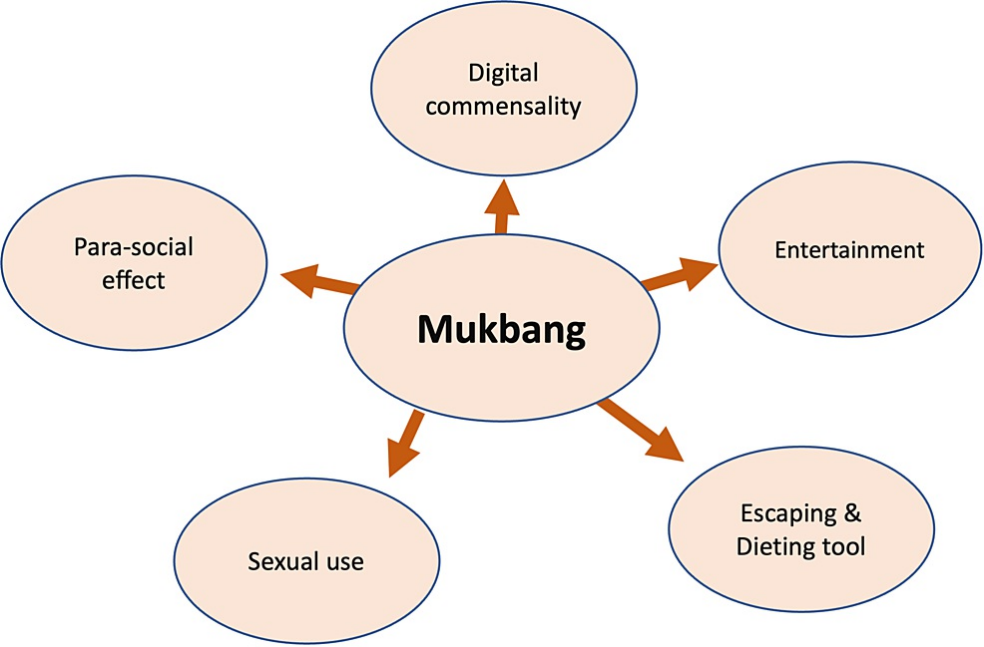
Diagrammatic representation of motivations behind watching mukbang videos

The effects of these videos on their audience need to be researched in order to gain an understanding of the reasons why people spend time watching mukbang videos. This time spent watching mukbang videos can range from watching a single video to watching multiple videos in quick succession. Figure 1 below shows the various motivations behind watching mukbang videos.

Many studies have shown that there has been an increase in the number of people living alone, and this has led to an increase in the number of people watching mukbang as a means of being connected together as it is a way for viewers to virtually get eating partners [9]. As a result, mukbang watching provides a sense of digital commensality, which refers to the feeling of being connected through digital technology-based intervention even when dining alone. As a result, people feel united with each other simply by seeing someone else eating online [9,26]. In general, according to the findings of the vast majority of studies that have been conducted up to this point, the fact that watching mukbang can help viewers feel less isolated is one of the primary factors contributing to mukbang's expanding popularity all over the world [7-9,14,16,17,19-21]. According to the findings of a study that took a netnographic approach, people often consider the hosts of mukbang videos to be their "meal mates" while they are dining alone in order to alleviate the feelings of seclusion that come along with the experience [23]. In addition to this, mukbang watching provides the viewers with a sense of co-presence as a result of the comments and likes that are typed by the host as well as the co-viewers while they are watching the videos of people eating food [9].

A para-social relationship, which is formed between the host videos and the audience upon the repeated watching of mukbang videos, is another term that has been used in the netnographic study [23,30]. This term is both old and interesting. This para-social relation has expanded the interpersonal relationship of the individuals watching the videos, in addition to reducing the lonesomeness of the individuals who are watching the videos [23]. The viewers develop a feeling of emotional relation and empathy with the mukbangers, which can be attributed to the fact that the mukbang host usually shares their personal life experiences or their daily experiences with the audience, which gives the viewers a sense of community. The viewers develop a sense of emotional relation and empathy with the mukbangers [6,7,9,30].

It has also been discovered that watching mukbang can be a great source of entertainment. In particular, the gustatory expressions that the host makes while chewing, gulping, or even the sound of opening a soda can or a packet of food can provide the viewers with a variety of gratifications [6-9,13,22,31]. These expressions include "mmm," "ymm," and "yuck." Additionally, because of this, viewers are compelled to watch mukbang multiple times before they feel satisfied. These sounds also elicited an autonomous sensory meridian response (ASMR), which provides the audience with a sense of relief, relaxation, delight, and sedative effect [9,21,23,32,33]. The heightened pleasure that is displayed by mukbangers while they are eating the food in the videos can result in a decrease in the pleasure that is experienced by the viewers when eating food in real life, which can contribute to eating disorders [23]. These kinds of effects can cause viewers to eat less of the actual food, which puts an individual at an increased risk of developing anorexia nervosa [9,34].

Many mukbangers also include cooking videos of the foodstuffs that they are going to have. All of this, along with some tips on cooking that are included in the video, makes these videos even more interesting and entertaining for the spectators to watch [28].

The viewers also used to watch mukbang as a means of evading the unappealing or unpleasant aspects of day-to-day life by distracting themselves with the activity [35]. For example, if a person is unable to consume the food they want because it is not readily available, they can find solace in the fact that they can watch a video of someone else eating the food online and get the same satisfaction [35]. Mukbang also serves as a form of escape for viewers by diverting their attention away from the challenges they face in their personal lives whenever the host discusses their day-to-day lives [9]. People are able to reduce the amount of stress in their lives by doing so [8,9,15]. One of the studies found that when people watch someone else eat food on camera, it makes them feel as if they are eating the food themselves. They can almost sense the flavor of the food, and they have a feeling of satiety [9,21,23,32]. Due to the fact that many viewers watch mukbangers in order to experience the pleasure of eating vicariously through ASMR despite the fact that they are on a diet, it has been determined that watching mukbang can be considered a "diet tool" [7,36]. The phenomenon of vicarious satiation, in which the viewers' desire to eat specific foods is affected by watching the mukbangers eating the same food, has been called a magical eating fantasy, which means that the person can eat as much food as he wants without even suffering from the bad consequences of doing so. Multiple experiments conducted by different studies have revealed the phenomenon of vicarious satiation [7,9,17]. Some viewers watch mukbang in place of eating so that they can have the experience of eating while also satisfying their desire to eat. In this way, they can "fulfill their fantasies of eating" [7,9,17].

Mukbang is known to have some potential for sexual connotations as well. Previous research has shown that overweight male viewers of mukbang were motivated to do so by a desire to fantasize about thin, attractive female mukbangers gorging themselves on large quantities of food [9,36-38]. In 2018, researchers reported that mukbang may be leading to a fetishization of the eating behaviors of women, which they attributed to the phenomenon [9]. In addition, they comment that the women in mukbang videos typically demonstrate an embarrassingly large appetite, which is something that women try to hide most of the time [9]. This kind of portrayal has the potential to sexualize the physical characteristics of women. The researchers are concerned that through mukbang there exists such potential objectification of women's bodies from a primarily sexual aspect and that it strengthens the stereotyping of women as possessing "thinness" for consumerism [9]. This has been shown to be a concern by the researchers. In an earlier study conducted by Donnar, the researchers came to the conclusion that thin and attractive women who participated in mukbang videos typically had overweight men as their primary audience for the videos [36]. In mukbang, the women are shown consuming enormous amounts of food, which creates a vulnerable and private setting [9]. According to Donnar, this makes it easier to cast a sexualized gaze toward the women who are involved [36]. In addition, he came to the conclusion that the sexual setting and eating sensations provided by the mukbang videos frequently left the audience with a range of feelings, such as yearning, disgust, pleasure, shame, envy, and desire [36]. The phenomenon of mukbang has only been subjected to critical analysis in a very limited number of recent studies. An investigation into the motivations behind watching mukbang was carried out in 2019 as part of a cross-cultural study that involved the participation of 243 people who belonged to either the Asian or the Caucasian ethnic groups [9]. According to the findings of the study, there is a positive connection between the attractiveness of the host and attitudes toward mukbang held by the two ethnic groups [9]. In addition, having a large audience is related not only to the host's physical attractiveness but also to their sociability and their general likeability [9].

The enormous amount of food that is shown to be consumed by the thin host during mukbang, despite the fact that it somehow normalizes the fact that eating large portions of food (binge eating) is normal while still maintaining a lean body mass and remaining healthy, is one of the challenging aspects of mukbang. This has led many of the viewers of mukbang to have unrealistic beliefs, which has resulted in a significant increase in the amount of food they consume [9,37,39]. This can ultimately lead to negative results such as obesity, and it can also oftentimes trigger disordered eating behaviors [9,18].

One more thing that has come to people's attention is the fact that the food that is typically displayed in the mukbang is low-cost, unhealthy, frozen instant meals that have very little nutritional value in them [9,40,41]. This may make viewers less interested in eating home-cooked meals and increase the popularity of brands of junk food and unhealthy frozen foods among these consumers [9,42-44]. Studies have shown that people whose diets were not monitored while watching mukbang had an increase in the amount of food they consumed as a result of the activity. While this may be advantageous for some people (solely in the sense that it will cause them to consume more food, but not in the sense that it will improve their nutrition), it may be detrimental for others because it will cause them to eat more food than they would normally and, as a result, more calories than their bodies require [9,45]. When viewers themselves struggle with some form of eating disorder, the negative effects of their problematic eating are amplified to a much greater degree. In addition to this, it has led to a variety of disorders that are associated with the consumption of an excessive amount of food, such as the rise in the prevalence of obesity and eating disorders such as anorexia nervosa, bulimia nervosa, orthorexia nervosa, and a great number of other eating disorders [18,46]. The poor table manners displayed in mukbang videos, such as eating food sloppily, stuffing one's entire face with food, and talking while eating, also have a negative impact on young adults and can have damaging long-term consequences [47,48]. Due to the fact that viewing mukbang can have such a wide variety of effects on its audience, it is of the utmost importance for researchers and clinicians to be aware of the effects that mukbang can have [13].

## Conclusions

Nevertheless, this review paper sheds light on the myriad of motivations behind watching mukbang as well as its health effects on its viewers. At this point, mukbang is regarded as a bipartite phenomenon, but a clear line cannot yet be drawn dividing the positive and negative aspects of mukbang in a person's life. In light of the fact that there is only a limited amount of research currently available on mukbang, there is a pressing need to place a greater emphasis on the phenomenon of mukbang, and clinicians should be made aware of mukbang in order to facilitate the diagnosis of conditions that are linked to mukbang.
